# Prognostic Significance of Skin Toxicity in Patients with Ras Wild-Type Metastatic Colorectal Cancer Treated with Anti-Egfr Monoclonal Antibodies

**DOI:** 10.3390/jcm15093214

**Published:** 2026-04-23

**Authors:** Ridvan Gonul, Oktay Bozkurt, Gozde Erturk Zararsiz, Bugra Umut Kaya, Ahmet Kursat Disli, Ugur Turkmen, Ayse Nuransoy Cengiz, Muhammet Cengiz, Kamuran Yuceer, Mevlude Inanc, Metin Ozkan

**Affiliations:** 1 Department of Medical Oncology, Faculty of Medicine, Erciyes University, 38039 Kayseri, Turkey; ridvangonull@gmail.com (R.G.); kdisli@gmail.com (A.K.D.); ugrmen38@gmail.com (U.T.); aysenuransoy@hotmail.com (A.N.C.); drkamuranaltiparmak@gmail.com (K.Y.); mevludeinanc@hotmail.com (M.I.); metino@erciyes.edu.tr (M.O.); 2 Department of Biostatistics, School of Medicine, Erciyes University, 38039 Kayseri, Turkey; 3 Department of Internal Medicine, Faculty of Medicine, Erciyes University, 38039 Kayseri, Turkey; 4 Department of Medical Oncology, Kayseri City Hospital, 38080 Kayseri, Turkey; mhmmtcengiz@hotmail.com

**Keywords:** skin toxicity, metastatic colorectal cancer, prognosis, anti-EGFR monoclonal antibodies, RAS wild-type

## Abstract

**Background and Aim:** Anti-epidermal growth factor receptor (EGFR) therapy is commonly associated with skin toxicity, which may reflect treatment response. This study evaluated the prognostic significance of anti-EGFR-related skin toxicity in patients with RAS wild-type metastatic colorectal cancer (mCRC) receiving palliative chemotherapy. **Materials and Methods:** We retrospectively analyzed 256 RAS wild-type mCRC patients treated with anti-EGFR monoclonal antibodies at Erciyes University, Kayseri, Turkey (June 2011–February 2024). Survival was estimated using the Kaplan-Meier method with log-rank comparisons. A landmark analysis at 2 months was performed to address guarantee-time bias. Univariate and multivariate Cox regression analyses were used to identify independent prognostic factors. **Results:** The median PFS was 17 months in patients with grade ≥ 2 skin toxicity versus 8 months in those with grade < 2 skin toxicity (*p* < 0.001). The median OS was 32 and 21 months, respectively (*p* < 0.001). In the landmark-adjusted multivariate analysis, grade ≥ 2 skin toxicity was an independent prognostic factor for both PFS (HR 0.52, 95% CI 0.39–0.70, *p* < 0.001) and OS (HR 0.50, 95% CI 0.37–0.68, *p* < 0.001). Additional independent factors for OS included albumin, LDH, peritoneal metastasis, age, tumor sidedness, and BMI. The objective response rates were 53.9% and 11.3% in the grade ≥ 2 and grade < 2 groups, respectively (*p* < 0.001). **Conclusions:** Grade ≥ 2 skin toxicity was significantly associated with longer PFS, OS, and a higher response rate, and was confirmed as an independent prognostic factor in multivariate analysis. These findings suggest that skin toxicity may serve as a non-invasive marker of treatment efficacy. Prospective studies with time-dependent methodologies are needed to validate these results.

## 1. Introduction

According to the World Health Organization GLOBOCAN data, colorectal cancer (CRC) remains the third most commonly diagnosed malignancy in men and the second most commonly diagnosed malignancy in women worldwide [1]. Approximately 15–30% of patients present with metastatic disease at the time of diagnosis, while 20–50% of those with initially localized disease subsequently develop metastases. The most frequent site of metastasis is the liver, followed by the lung, peritoneum, and distant lymph nodes [2].

The biology of metastatic colorectal cancer (mCRC) has become increasingly well understood in recent years, and this knowledge is directly shaping treatment decisions. RAS (KRAS and NRAS) mutations are detected in approximately 40–50% of mCRC patients and are strongly associated with resistance to anti-EGFR therapies [2,3]. The BRAF V600E mutation is observed in 8–12% of patients and is associated with poor prognosis; in this subgroup, combinations of BRAF and MEK inhibitors (encorafenib + binimetinib + cetuximab) have become the standard of care [2]. Deficient mismatch repair (dMMR)/high microsatellite instability (MSI-H) is present in fewer than 5% of mCRC patients; however, immune checkpoint inhibitors (pembrolizumab, nivolumab) have demonstrated dramatic response rates in this subgroup and are now accepted as first-line standard therapy [2,4]. Primary tumor location is also a determining factor in treatment selection, with left-sided tumors deriving substantially greater benefit from anti-EGFR therapies [2].

In patients with RAS and BRAF wild-type tumors, anti-epidermal growth factor receptor (EGFR) monoclonal antibodies—cetuximab and panitumumab—provide substantial survival advantages when added to FOLFOX or FOLFIRI regimens in the first- and second-line settings [2,3]. Although chemo-immunotherapy combinations are being investigated in these patients, the role of immunotherapy in mCRC currently remains limited to the dMMR/MSI-H subgroup. However, anti-EGFR therapies are associated with notable adverse effects. EGFRs expressed in normal human skin tissue play a role in keratinocyte proliferation, cell growth, and production of the outer root sheath of hair follicles. Consequently, various cutaneous reactions, including acneiform rash, paronychia, and dry skin, may occur in 45–100% of patients and constitute the most common adverse effect of EGFR inhibitor therapy [5,6].

Skin toxicity (ST) has attracted attention not only as a side effect but also as a potential marker of treatment efficacy. Although multiple retrospective analyses and randomized studies have reported that pronounced cutaneous reactions are associated with OS and PFS, some studies have failed to confirm this association; thus, the topic remains a matter of debate [7,8,9,10,11]. The underlying mechanism by which skin toxicity reflects treatment response has not been fully elucidated; however, it is thought to indicate effective inhibition of the EGFR signaling pathway. In this context, the question of whether skin toxicity can serve as a clinically useful, non-invasive prognostic tool remains relevant.

The primary research question of this study is as follows: Is the development of grade ≥ 2 skin toxicity an independent prognostic factor for PFS and OS in patients with RAS wild-type mCRC receiving anti-EGFR therapy? In the existing literature, this association has been reported with inconsistent results and has not been adequately evaluated alongside other prognostic variables in multivariate analyses. This study aims to systematically examine the prognostic value of anti-EGFR-related skin toxicity in a cohort of 256 patients spanning a 13-year observation period.

## 2. Methods

### 2.1. Patient Data

Between June 2011 and February 2024, 256 patients with RAS wild-type mCRC treated with palliative therapy were included in this single-center retrospective study. The study was approved by the Ethics Board of Erciyes University Medical School, Melikgazi/Kayseri, Turkey.

The inclusion criteria were as follows: (a) patients with confirmed histopathologic stage IV CRC, (b) patients receiving anti-EGFR therapy in combination with chemotherapy, (c) patients with complete clinical records, including demographics, pathology, and treatment modalities, and (d) patients with Eastern Cooperative Oncology Group (ECOG) performance status of 0, 1, or 2.

The exclusion criteria were as follows: (a) patients who discontinued treatment due to intolerance to therapy or who died early during treatment, (b) patients with a documented second malignant tumor at a different location.

Patient records were retrospectively accessed through an electronic medical record system. Collected data included patient demographics, tumor location, histopathological findings, molecular analysis results (RAS/BRAF mutation status), metastatic sites, chemotherapy and anti-EGFR regimen administered, treatment line, laboratory parameters (albumin, LDH, CEA), and treatment response assessments.

### 2.2. Skin Toxicity Assessment

Skin toxicity was assessed at each chemotherapy/anti-EGFR treatment cycle using the National Cancer Institute Common Terminology Criteria for Adverse Events (NCI-CTCAE) version 4.0. Although the study period spanned 13 years (2011–2024), the NCI-CTCAE v4.0 criteria were consistently applied throughout the grading process. Patients were classified into two groups: clinically non-relevant skin toxicity (grade < 2) and clinically relevant skin toxicity (grade ≥ 2). Most patients received prophylactic skin care management, which included topical corticosteroids, emollients, sunscreen, and oral doxycycline when clinically indicated. The decision to initiate and the intensity of prophylactic measures were at the discretion of the treating physician.

### 2.3. Data Collection and Imaging

Pre-chemotherapy blood analyses, including complete blood count and blood chemistry, were performed 2–24 h before treatment administration. Biochemical analyses were performed using a Roche Cobas c 702 (Roche Diagnostics, Mannheim, Germany) device. All patients underwent pre-chemotherapy staging with computed tomography (CT) of the abdomen and thorax. Additional imaging modalities, such as magnetic resonance imaging (MRI), positron emission tomography (PET), and bone scintigraphy, were performed based on the patients’ symptoms or at the discretion of the treating physician. Baseline CT imaging was obtained before treatment initiation, and follow-up imaging was performed every 8–12 weeks thereafter. Radiological response was assessed according to the Response Evaluation Criteria in Solid Tumors (RECIST). The objective response rate (ORR) was defined as the proportion of patients who achieved a complete or partial response based on RECIST.

### 2.4. Statistical Analysis

The Shapiro-Wilk test, histogram, and Q-Q plots were used to assess the distribution of the data. Differences between groups were compared using Fisher’s exact test and Pearson’s χ^2^ test for categorical variables. Values are expressed as frequency and percentage, mean and standard deviation, or median and minimum-maximum. Progression-free survival (PFS) was calculated as the time from the start of the first anti-EGFR treatment to the date of progression in months. Overall survival (OS) was defined as the interval from the first anti-EGFR treatment to the date of death or the last follow-up in months. Risk factors for PFS and OS were evaluated using univariate and multivariate Cox proportional hazards regression analyses. To minimize guarantee-time bias related to the development of skin toxicity, a landmark analysis was performed at 2 months after treatment initiation, including only patients who were alive and progression-free at this time point, and survival times were recalculated from the landmark. Patients who experienced disease progression or died before the 2-month landmark were excluded from this analysis. Variables were selected using a backward stepwise Cox regression model based on likelihood ratio testing, and anti-EGFR-related skin toxicity was forced into the multivariate model regardless of statistical significance. The Kaplan-Meier method was applied to estimate PFS and OS, and the log-rank test was performed for group comparisons. The hazard ratio was obtained with 95% confidence intervals, and a *p*-value < 0.05 was considered statistically significant. Statistical analyses were performed using R (version 4.4.2) and SPSS Statistics (version 27.0; IBM Corp., Armonk, NY, USA).

## 3. Results

The study included 256 patients with mCRC, of whom 165 (64.5%) were men and 91 (35.5%) were women. The median age of the patients was 63 years (25–90 years). The tumor was located in the right colon in 50 patients (19.5%) and the left colon in 206 patients (80.5%). The most common site of metastasis was the liver in 172 (67.2%) patients, followed by the lung in 69 (27%) and the peritoneum in 29 (11.3%) patients. A total of 214 patients (83.6%) received anti-EGFR monoclonal antibody therapy as first-line treatment, and 42 patients (16.4%) received it as second-line treatment.

Patients were grouped according to clinically non-relevant anti-EGFR-ST (grade < 2) and clinically relevant anti-EGFR-ST (grade ≥ 2).

The baseline clinicopathological and treatment-related characteristics of the 256 patients according to anti-EGFR-ST groups are presented in Table 1. All baseline variables, including metastatic sites, were recorded at the time of anti-EGFR treatment initiation, and treatment response was assessed during follow-up. Peritoneal metastasis was significantly less frequent in the grade ≥ 2 group (6.1% vs. 15.6%, *p* = 0.018), and a higher proportion of patients in this group received oxaliplatin-based chemotherapy (49.6% vs. 33.3%, *p* = 0.01). The objective response rate was markedly higher in the grade ≥ 2 group (53.9% vs. 11.3%, *p* < 0.001). No significant differences were observed between groups regarding age, sex, ECOG performance status, tumor sidedness, anti-EGFR agent type, treatment line, or laboratory parameters.

Table 2 presents the results of the landmark-adjusted univariate and multivariate Cox regression analyses at 2 months for PFS. In the univariate analysis, anti-EGFR-ST grade ≥ 2 (HR: 0.49 (0.37–0.66); *p* < 0.001), albumin levels < 4 g/dL (HR: 1.64 (1.14–2.37); *p* = 0.008), ECOG performance status 2 (HR: 1.35 (0.97–1.87); *p* = 0.075), and the presence of comorbidity (HR: 0.80 (0.60–1.08); *p* = 0.150) were identified as significantly associated with survival outcomes. Conversely, no significant associations (*p* ≥ 0.250) were observed for age, sex, primary tumor site, chemotherapy backbone, anti-EGFR agent type, peritoneal metastasis, LDH levels, CEA levels, or BMI in the univariate model. The multivariate landmark model confirmed that grade ≥ 2 skin toxicity remained a robust and independent prognostic factor for superior PFS, demonstrating a 48% reduction in the risk of progression compared with the grade < 2 group (HR: 0.52 (0.39–0.70), *p* < 0.001). Notably, although albumin was significant in the univariate analysis, it did not reach independent statistical significance in the multivariate landmark model (*p* = 0.094).

Table 3 presents the landmark-adjusted univariate and multivariate Cox regression analyses at 2 months for OS. In the univariate analysis, anti-EGFR-ST grade ≥ 2 (HR 0.49, 95% CI 0.37–0.65, *p* < 0.001), peritoneal metastasis (HR 2.00, 95% CI 1.29–3.10, *p* = 0.002), LDH ≥ ULN (HR 1.37, 95% CI 1.03–1.83, *p* = 0.029), albumin < 4 g/dL (HR 1.84, 95% CI 1.28–2.63, *p* < 0.001), BMI ≥25 (HR 0.68, 95% CI 0.51–0.91, *p* = 0.009), and ECOG PS 2 (HR 1.41, 95% CI 1.03–1.94, *p* = 0.034) were significantly associated with OS. In the multivariate model, independent prognostic factors for OS were: anti-EGFR-ST grade ≥ 2 (HR 0.50, 95% CI 0.37–0.68, *p* < 0.001), albumin (HR 1.56, 95% CI 1.08–2.25, *p* = 0.018), LDH (HR 1.45, 95% CI 1.08–1.95, *p* = 0.013), peritoneal metastasis (HR 1.58, 95% CI 1.01–2.47, *p* = 0.043), age ≥ 65 (HR 0.71, 95% CI 0.52–0.96, *p* = 0.025), left-sided tumor location (HR 0.58, 95% CI 0.40–0.84, *p* = 0.004), and BMI ≥ 25 (HR 0.69, 95% CI 0.51–0.93, *p* = 0.014).

**Figure 1:**
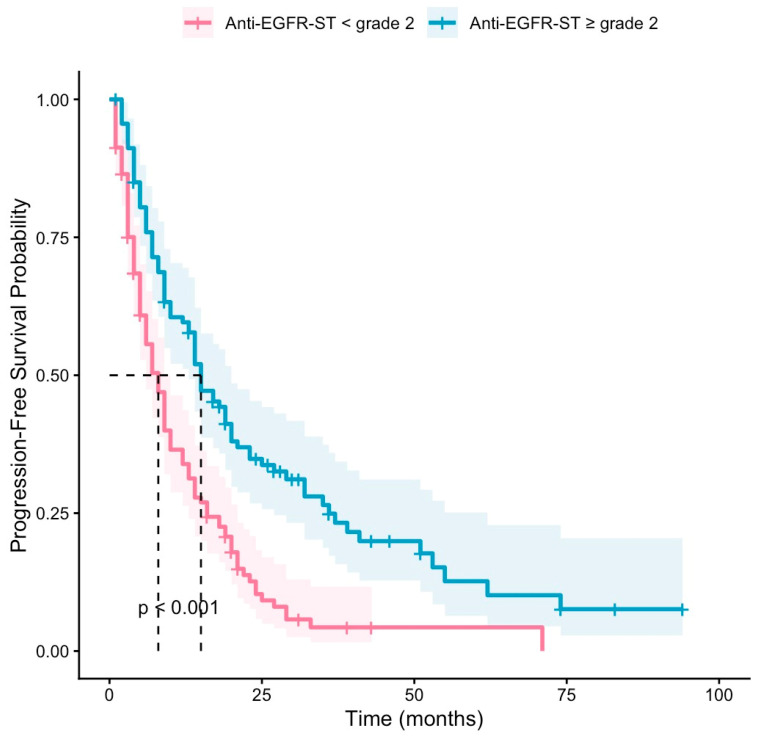
Kaplan-Meier curves of PFS according to anti-EGFR-ST groups. Shaded areas represent 95% confidence intervals. Dashed lines indicate median survival times. Tick marks represent censored observations.

**Figure 2:**
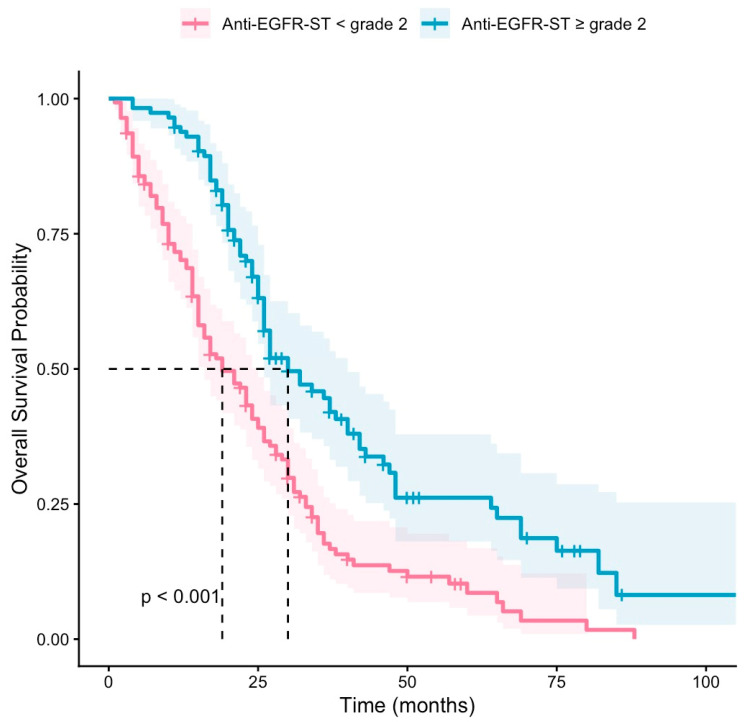
Kaplan–Meier curves of OS according to anti-EGFR-ST groups. Shaded areas represent 95% confidence intervals. Dashed lines indicate median survival times. Tick marks represent censored observations.

The objective remission rate (ORR) was 53.9% in the anti-EGFR-ST grade ≥ 2 group, while it was 11.3% in the anti-EGFR-ST grade < 2 group (*p* < 0.001). The median PFS was 8 months in the anti-EGFR-ST grade < 2 (95% CI: 6.24–9.75) and 17 months (95% CI: 13.98–20.01) in the anti-EGFR-ST grade ≥ 2 group (*p* < 0.001) (Figure 1). The median OS was 21 months (95% CI: 16.24–25.75) in the anti-EGFR-ST grade < 2 group and 32 months (95% CI: 24.19–39.80) in the anti-EGFR-ST grade ≥ 2 group (*p* < 0.001) (Figure 2).

## 4. Discussion

Patients treated with EGFR inhibitors frequently develop an acneiform rash consisting of inflammatory papules and pustules on the face, neck, scalp, and upper trunk. The frequency of cutaneous toxicity ranges from 50% to 100%, depending on the agent used and the type of cancer, and the rash typically appears within 1–2 weeks after treatment initiation [11]. In this study, we retrospectively evaluated the prognostic impact of grade ≥ 2 skin toxicity on PFS, OS, and ORR in RAS wild-type mCRC patients treated with anti-EGFR therapy.

The central finding of this study was that patients who developed grade ≥ 2 skin toxicity had significantly longer median PFS (17 vs. 8 months, *p* < 0.001) and median OS (32 vs. 21 months, *p* < 0.001) than those with grade < 2 toxicity. These results are consistent with those of several published studies. Tougeron et al. demonstrated that patients with grade ≥ 2 skin reactions had significantly longer median PFS and OS compared to those with grade < 2 reactions [8]. Holch et al., in a post-hoc analysis of the FIRE-3 trial, showed that cetuximab-induced grade ≥ 2 skin toxicity was positively associated with favorable outcomes in mCRC [12]. Peeters et al. also reported that among chemorefractory mCRC patients receiving panitumumab monotherapy, those with more severe skin toxicity had significantly longer OS (grade ≥ 2 vs. grade < 2; HR 0.60, *p* = 0.0033) [13]. However, Chiang et al. emphasized that this association was not consistent across all patient subgroups and suggested that the prognostic value of skin toxicity warrants further investigation [10]. Our study contributes to this ongoing debate by providing multivariate landmark-adjusted data in a relatively large cohort of 256 patients.

Regarding ORR, the grade ≥ 2 skin toxicity group achieved a rate of 53.9% compared to only 11.3% in the grade < 2 group (*p* < 0.001). This finding is highly concordant with the study by Cunningham et al., who reported a response rate of 55.2% in mCRC patients with severe skin reactions to cetuximab versus 6.3% in those without [14]. Orditura et al. similarly reported a strong correlation between skin rash and treatment efficacy [15]. Taken together, these findings support the hypothesis that skin toxicity may reflect effective inhibition of the EGFR signaling pathway and therefore could serve as a surrogate marker of tumor response.

A key methodological concern in studies evaluating on-treatment toxicity as a prognostic factor is guarantee-time bias, as patients must survive long enough to develop the exposure of interest. To address this limitation, we performed a landmark analysis at 2 months after treatment initiation, excluding patients who progressed or died before this time point. In the landmark-adjusted multivariate analysis, grade ≥ 2 skin toxicity remained a robust independent prognostic factor for both PFS (HR 0.52, 95% CI 0.39–0.70, *p* < 0.001) and OS (HR 0.50, 95% CI 0.37–0.68, *p* < 0.001). The consistency of the results between the primary and landmark-adjusted analyses strengthens the validity of the observed association.

The landmark-adjusted multivariate analysis for OS identified several additional independent prognostic factors. Peritoneal metastasis was associated with worse OS (HR 1.58, *p* = 0.043), consistent with the known poor prognosis of this subgroup. Franko et al. reported that peritoneal metastasis was detected in approximately 13% of CRC patients and that median survival in this group was shorter than in those with liver metastases only [16]. Elevated LDH was also an independent unfavorable prognostic factor for OS (HR 1.45, *p* = 0.013). Malignant cells rely extensively on aerobic glycolysis, known as the Warburg effect [17,18], and LDH plays a central role in this pathway [19]. Elevated LDH levels have been consistently associated with worse survival across multiple cancer types [20]. Low albumin, a negative acute phase reactant reflecting nutritional status, was similarly confirmed as an independent prognostic factor for OS (HR 1.56, *p* = 0.018), in line with previous reports in mCRC [21].

Notably, three additional variables emerged as significant in the landmark-adjusted OS model, which were not significant in the primary analysis. Older age (≥65 years) was associated with better OS (HR 0.71, *p* = 0.025), which may reflect selection bias in this retrospective cohort, as elderly patients who tolerated anti-EGFR therapy and survived beyond the 2-month landmark may represent a biologically favorable subgroup. Left-sided tumor location was independently associated with better OS (HR 0.58, *p* = 0.004), consistent with the well-established prognostic advantage of left-sided tumors in mCRC treated with anti-EGFR agents [2]. Higher BMI (≥25 kg/m^2^) was also associated with improved OS (HR 0.69, *p* = 0.014). Although the obesity paradox in cancer remains debated, several studies have reported a survival advantage associated with a higher BMI in patients with mCRC [22].

Most patients in this study received prophylactic skin care management including topical corticosteroids, emollients, sunscreen, and oral doxycycline when clinically indicated. This practice may have attenuated the overall incidence and severity of skin toxicity, potentially reducing the proportion of patients classified in the grade ≥ 2 group. Nevertheless, the fact that patients who developed clinically significant skin toxicity despite prophylactic measures had substantially better survival outcomes further supports the prognostic relevance of this finding.

### 4.1. Limitations

This study has several important limitations. First, its retrospective design and single-center origin in Turkey may limit the generalizability of the results. Second, because skin toxicity is an on-treatment event rather than a baseline variable, the analysis is inherently vulnerable to guarantee-time bias; this limitation was addressed through a landmark analysis at 2 months, although residual bias cannot be excluded entirely. Third, not all potential confounding factors influencing skin toxicity—such as individual skin care habits, sun exposure, and genetic polymorphisms—could be assessed. Fourth, the prophylactic skin care measures administered to most patients may have altered the incidence and severity of observed skin toxicity. Fifth, this study included only patients treated with anti-EGFR-based therapy; therefore, it can demonstrate an association between on-treatment toxicity and outcomes but cannot establish predictive utility in the strict sense, as no comparator arm without anti-EGFR therapy was included. Finally, the 13-year study period may have introduced heterogeneity due to evolving treatment protocols and supportive care practices over time.

### 4.2. Conclusions

The present data demonstrate that anti-EGFR-related skin toxicity of grade ≥ 2 is an independent prognostic factor for PFS and, together with LDH, peritoneal metastasis, albumin, age, tumor sidedness, and BMI, for OS in RAS wild-type mCRC patients. These findings suggest that skin toxicity may be considered not only an adverse event but also as a non-invasive marker of treatment efficacy in clinical practice. However, the present data are hypothesis-generating in nature. Larger prospective studies incorporating time-dependent methodologies and comparator arms are warranted to validate the prognostic and predictive potential of anti-EGFR-related skin toxicity in mCRC.

## Figures and Tables

**Table 1:** Baseline clinicopathological characteristics of participants according to anti-EGFR-ST groups.

Variables	Anti-EGFR-ST Group		Total (*n* = 256)	*p*-Value
	Grade < 2 (*n* = 141)	Grade ≥ 2 (*n* = 115)		
Gender				
Female	54 (38.3)	37 (32.2)	91 (35.5)	**0.359**
Male	87 (61.7)	78 (67.8)	165 (64.5)	
Age (years)				
<65	81 (57.4)	74 (64.3)	155 (60.5)	0.304
≥65	60 (42.6)	41 (35.7)	101 (39.5)	
ECOG performance status				
0–1	108 (76.6)	96 (83.5)	204 (81.9)	0.212
2	33 (23.4)	19 (16.5)	52 (18.1)	
Metastatic site				
Liver	92 (65.2)	80 (69.6)	172 (67.2)	0.505
Lung	37 (26.2)	32 (27.8)	69 (27)	0.779
Peritoneum	22 (15.6)	7 (6.1)	29 (11.3)	**0.018**
Tumors site				
Right colon	24 (17)	26 (22.6)	50 (19.5)	0.272
Left colon	117 (83)	89 (77.4)	206 (80.5)	
Chemotherapy				
FOLFIRI ^a^	94 (66.7)	58 (50.4)	152 (59.4)	**0.01**
Oxaliplatin based ^b^	47 (33.3)	47 (49.6)	104 (40.6)	
Anti-EGFR therapy				
Panitumumab	46 (32.6)	50 (43.5)	96 (37.9)	0.092
Cetuximab	95 (67.4)	65 (56.5)	160 (62.1)	
Anti-EGFR Therapy Line				
First-Line	112 (79.4)	102 (88.7)	214 (83.6)	0.061
Second-Line	29 (20.6)	13 (11.3)	42 (16.4)	
Lactate dehydrogenase (LDH) ^c^				
<ULN	75 (53.2)	61 (53)	136 (53.1)	1.000
≥ULN	66 (46.8)	54 (47)	120 (46.9)	
Albumin				
<4 g/dL	28 (19.9)	14 (11.4)	42 (16.1)	0.086
≥4 g/dL	113 (80.1)	101 (88.6)	214 (83.6)	
CEA (ng/mL)				
<ULN	65 (46.1)	46 (40)	111 (43.4)	0.375
≥ULN	76 (53.9)	69 (60)	145 (56.6)	
BMI (kg/m^2^)				
<25	60 (42.6)	45 (39.1)	105 (41)	0.611
≥25	81 (57.7)	70 (60.9)	151 (59)	
Adjuvant chemotherapy				
Yes	81 (57.4)	70 (60.9)	151 (59)	0.611
No	60 (42.6)	45 (39.1)	105 (41)	
Treatment response				
CR + PR	16 (11.3)	62 (53.9)	78 (30.5)	<0.001
PD + SD	125 (88.7)	53 (46.1)	178 (69.5)	

Values are expressed as *n* (%). ^a^ Folfiri (Irinotecan + Leucovorin + Fluorouracil), ^b^ Xelox (Oxaliplatin + Capecitabine) or Folfox (Oxaliplatin + Leucovorin + Fluorouracil) ^c^ Upper limit of reference range: 250 U/L; ULN: Upper limit of normal; ECOG: Eastern Cooperative Oncology Group; CEA: Carcinoembryonic Antigen; BMI: Body Mass Index; CR: Complete Response; PR: Partial Response; SD: Stable Disease; PD: Progressive Disease. Statistically significant *p* values are shown in bold.

**Table 2:** Univariate and multivariate Cox regression analysis for PFS (landmark analysis at 2 months).

Variables	Univariate		Multiple	
	HR (95% CI)	*p*-Value	HR (95% CI)	*p*-Value
**Age (years)**				
<65	1.00	-	-	-
≥65	0.85 (0.64–1.14)	0.275		
**Gender**				
Female	1.00	-	-	-
Male	0.87 (0.65–1.16)	0.335		
**ECOG performance status**				
0–1	1.00	-	-	-
2	1.35 (0.97–1.87)	0.075		
**Chemotherapy**				
FOLFIRI ^a^	1.00	-	-	-
Oxaliplatin based ^b^	1.00 (0.75–1.32)	0.975		
**Anti-EGFR therapy**				
Panitumumab	1.00	-	-	-
Cetuximab	1.08 (0.81–1.44)	0.603		
**Peritoneal metastasis**				
No	1.00	-	-	-
Yes	1.08 (0.81–1.44)	0.283		
**Lactate dehydrogenase (LDH) ^c^**				
<ULN	1.00	-	-	-
≥ULN	1.28 (0.81–2.02)	0.528		
**Albumin**				
≥4 g/dL	1.00	-	1.00	-
<4 g/dL	1.64 (1.14–2.37)	0.008	1.64 (1.14–2.37)	**0.094**
**Anti-EGFR-ST**				
Grade < 2	1.00	-	1.00	**-**
Grade ≥ 2	0.49 (0.37–0.66)	<0.001	0.52 (0.39–0.70)	**<0.001**
**Tumors site**				
Right colon	1.00	-	-	-
Left colon	0.89 (0.63–1.27)	0.525		
**CEA (ng/mL) ^d^**				
<ULN	1.00	-	-	-
≥ULN	1.04 (0.78–1.38)	0.790		
**BMI (kg/m^2^)**				
<25	1.00	-	-	-
≥25	0.87 (0.65–1.16)	0.329		
**Comorbidity**				
No	1.00	-	1.00	-
Yes	0.80 (0.60–1.08)	0.150	0.78 (0.58–1.06)	0.113

CI: Confidence interval; HR: Hazard ratio; PFS: Progression-free survival; Anti-EGFR-ST: Anti-epidermal growth factor receptor-related skin toxicity; CEA: Carcinoembryonic antigen; BMI: Body mass index; ECOG performance status: Eastern Cooperative Oncology Group performance status; ^a^ Folfiri (Irinotecan + Leucovorin + Fluorouracil), ^b^ Xelox (Oxaliplatin + Capecitabine) or Folfox (Oxaliplatin + Leucovorin + Fluorouracil); ^c^ Upper limit of reference range: 250 U/L; ^d^ Upper limit of reference range: 6.5 ng/mL; ULN: Upper limit of normal; Statistically significant *p* values are shown in bold.

**Table 3:** Univariate and multivariate Cox regression analysis for OS (landmark analysis at 2 months).

Variables	Univariate		Multiple	
	HR (95% CI)	*p*-Value	HR (95% CI)	*p*-Value
**Age (years)**				
<65	1.00	-	1.00	-
≥65	0.77 (0.57–1.03)	0.076	0.71 (0.52–0.96)	**0.025**
**Sex**				
Female	1.00	-	-	-
Male	0.93 (0.70–1.25)	0.651		
**ECOG performance status**				
0–1	1.00	-	1.00	-
2	1.41 (1.03–1.94)	0.034	1.11 (0.79–1.56)	0.544
**Chemotherapy**				
FOLFIRI ^a^	1.00	-	-	-
Oxaliplatin based ^b^	1.05 (0.79–1.40)	0.727		
**Anti-EGFR therapy**				
Panitumumab	1.00	-	-	-
Cetuximab	1.02 (0.76–1.37)	0.894		
**Peritoneal metastasis**				
No	1.00	-	1.00	**-**
Yes	2.00 (1.29–3.10)	0.002	1.58 (1.01–2.47)	**0.043**
**Lactate dehydrogenase (LDH) ^c^**				
<ULN	1.00	-	1.00	**-**
≥ULN	1.37 (1.03–1.83)	0.029	1.45 (1.08–1.95)	**0.013**
**Albumin**				
≥4 g/dL	1.00	-	1.00	**-**
<4 g/dL	1.84 (1.28–2.63)	<0.001	1.56 (1.08–2.25)	**0.018**
**Anti-EGFR-ST**				
Grade < 2	1.00	-	1.00	-
Grade ≥ 2	0.49 (0.37–0.65)	<0.001	0.50 (0.37–0.68)	**<0.001**
**Tumors site**				
Right colon	1.00	-	1.00	**-**
Left colon	0.75 (0.53–1.07)	0.114	0.58 (0.40–0.84)	**0.004**
**CEA (ng/mL) ^d^**				
<ULN	1.00	-	-	-
≥ULN	1.22 (0.92–1.62)	0.173		
**BMI (kg/m^2^)**				
<25	1.00	-	1.00	**-**
≥25	0.68 (0.51–0.91)	0.009	0.69 (0.51–0.93)	**0.014**
**Comorbidity**				
No	1.00	-	-	-
Yes	0.78 (0.58–1.04)	0.094		

CI: Confidence interval; HR: Hazard ratio; OS: Overall survival; ECOG performance status: Eastern Cooperative Oncology Group performance status; ^a^ Folfiri (Irinotecan + Leucovorin + Fluorouracil); ^b^ Xelox (Oxaliplatin + Capecitabine) or Folfox (Oxaliplatin + Leucovorin + Fluorouracil); ^c^ Upper limit of reference range: 250 U/L; ^d^ Upper limit of reference range: 6.5 ng/mL; ULN: Upper limit of normal; Statistically significant *p* values are shown in bold.

## Data Availability

The data presented in this study are not publicly available due to patient privacy and ethical restrictions. The data may be made available upon reasonable request to the corresponding author, subject to institutional and ethical approval.

## Abbreviations

The following abbreviations are used in this manuscript:

Anti-EGFR-ST	Anti-epidermal growth factor receptor-associated skin toxicity
BMI	Body mass index
BRAF	B-Raf proto-oncogene
CEA	Carcinoembryonic antigen
CI	Confidence interval
CR	Complete response
CRC	Colorectal cancer
CT	Computed tomography
dMMR	Deficient mismatch repair
ECOG	Eastern Cooperative Oncology Group
ECOG PS	Eastern Cooperative Oncology Group performance status
EGFR	Epidermal growth factor receptor
HR	Hazard ratio
LDH	Lactate dehydrogenase
mCRC	Metastatic colorectal cancer
MSI-H	Microsatellite instability-high
NCI-CTCAE	National Cancer Institute Common Terminology Criteria for Adverse Events
ORR	Objective response rate
OS	Overall survival
PD	Progressive disease
PFS	Progression-free survival
PM	Peritoneal metastasis
PR	Partial response
RAS	Rat sarcoma virus proto-oncogene
RECIST	Response Evaluation Criteria in Solid Tumors
SD	Stable disease
ULN	Upper limit of normal
